# Modernizing vascular services to meet the demands of a changing disease burden

**DOI:** 10.1093/bjs/znab080

**Published:** 2021-03-22

**Authors:** David A Russell

**Affiliations:** Leeds Institute for Clinical Trials Research, University of Leeds, Leeds, UK

The workload of a vascular surgeon is evolving, with a reduction in the prevalence of aortic and symptomatic carotid disease and an escalating burden of chronic limb-threatening ischaemia (CLTI) and lower limb wounds, associated with diabetes and an ageing population. It is estimated that nearly 15 per cent of US Medicare beneficiaries have a wound^1^ and wound care accounts for 2–4 per cent of European healthcare budgets^2^. From 2012–2017 the prevalence of chronic wounds increased in the UK by 70 per cent, the largest increase being experienced in arterial (244 per cent) and mixed arterial ulcers (355 per cent)^3^. Over two thirds of expenditure in wound care is consumed by the 30 per cent that fail to heal^3^.

Non-healing wounds, stalled in a chronic inflammatory phase, increase the risk of infection, hospitalization and amputation. Failure to identify the wound aetiology appropriately, institute appropriate specific therapies including compression or off-loading, and refer promptly to specialist services have been identified as a primary cause of delayed wound healing^4^. To this end it is pleasing to observe the proportion of wounds labelled as an ‘unspecified leg ulcer’ falling from 19 per cent to 9 per cent^3^^,^^4^.

There is significant variability in delivery of wound care assessment and management, with multiple pathways involving disparate groups of physicians, even within the same healthcare system. The push to improve outcomes for people with diabetes with expedited multidisciplinary care provision has led to an inequitable service for those without diabetes. There is a need to reform services to address such healthcare inequalities, and meet the demands of hard-to-reach groups.

So, what is the role of the vascular surgeon in improving outcomes in lower limb wounds? Whilst advanced training in wound care has not been a priority in the vascular surgery curriculum of most nations, the delivery of timely, appropriate vascular intervention remains key to the management of the leading causes of lower limb wounds. There have been welcome recent advances in guidance, not least the change in terminology from ‘critical limb ischaemia’ to ‘chronic limb-threatening ischaemia’. Finally, common sense has prevailed, recognizing that a one-size-fits-all definition of ‘adequate’ wound perfusion is clinically incorrect. The global vascular guidelines also provide a structured framework for revascularization decision making, emphasizing the need for objective assessments of perfusion^5^. The WIfI (Wound, Ischaemia, foot Infection) score is a clinically useful tool to aid clinical decision making and helps standardize data for comparative purposes^6^^,^^7^.

The second factor influencing vascular surgery is the identification of urgency in delivery of CLTI interventions. The Vascular Society of Great Britain and Ireland CLTI Quality Improvement Programme has identified targets from initial consultation to revascularization of 5 days and 14 days for inpatients and outpatients respectively^8^, supported by evidence that delays of more than 14 days are an independent risk factor for amputation, particularly in patients with diabetes^9^. Vascular centres that have developed new services to achieve this have reported significant reduction in major amputations^10^. Delivery of these targets requires a move away from traditional pathways of care. One positive outcome of the COVID pandemic, driven by the need to minimize hospital visits and length of stay, has been the introduction of daily urgent clinics (‘hot clinics’) as a point of access for face-to-face urgent consultations, and a move to an immediate (same day in some cases) revascularization strategy where possible. Streamlining pathways may have deleterious effects, for example by compromising medical optimization and reducing the use of cross-sectional imaging. It will therefore be critical to evaluate comprehensively the impact of these innovative pathways on patient outcomes.

The third challenge is ensuring prompt referral of patients into the service, provision of holistic care and optimal wound management. The NHS National Wound Care Strategy Programme has identified this as a key factor in improving wound outcomes, recommending that all wounds without immediate red flag symptoms are referred to ‘a clinician with advanced wound care capabilities/competencies working within a multidisciplinary team (MDT) system for assessment, diagnosis and treatment planning’^11^. All foot wounds, irrespective of diabetes status, should be assessed within 1 day and leg wounds within 14 days. The impact of this guidance on wound outcomes is awaited, but national level audit data suggest that earlier referral is associated with better healing^12^.

There is a strong clinical rationale for inter-disciplinary management of the patient with a chronic wound, but what role should the vascular surgeon play within the team? A skilled vascular clinician with detailed knowledge of both wound care and vascular assessment/interventions is pivotal. This may be a vascular surgeon, in combination with a podiatrist, tissue viability nurse or vascular nurse specialist to provide wound care expertise. The ‘toe and flow’ model of joint working between vascular surgery and podiatry has been promoted internationally for those with chronic foot wounds but will not suit those patients with wounds above the ankle. Models with a vascular nurse specialist providing both wound care and vascular expertise have also been reported to deliver excellent outcomes and may be more cost effective. In Belgium and Germany external peer review of services takes the form of formal credentialling of diabetic foot clinics^13^, and in the UK the benefit of regional peer review of services in improving regional outcomes and reducing variation has also been demonstrated^14^. However implemented, the MDT must have the necessary expertise within it, well defined pathways of care for access, step-up and step-down of patients, and regular audit of outcomes with appropriate quality-improvement strategies where necessary.

Vascular disease is changing and the key challenge facing patients, healthcare professionals and healthcare systems are wounds of the lower limb, diabetes and CLTI. Vascular surgeons are well placed to coordinate and deliver care to this population. However, the demands that these patients place on the healthcare system are unique and require the development of innovative and evidence-based interventions and pathways of care.

## Disclosure

The author declares no conflict of interest.
